# Strengths-Based Behavioral Intervention for Parents of Adolescents With Type 1 Diabetes Using an mHealth App (Type 1 Doing Well): Protocol for a Pilot Randomized Controlled Trial

**DOI:** 10.2196/resprot.9147

**Published:** 2018-03-13

**Authors:** Marisa E Hilliard, Sahar S Eshtehardi, Charles G Minard, Rana Saber, Debbe Thompson, Lefkothea P Karaviti, Yuliana Rojas, Barbara J Anderson

**Affiliations:** ^1^ Section of Psychology Department of Pediatrics Baylor College of Medicine and Texas Children's Hospital Houston, TX United States; ^2^ Dan L Duncan Institute for Clinical and Translational Research Baylor College of Medicine Houston, TX United States; ^3^ Department of Preventive Medicine Feinberg School of Medicine Northwestern University Chicago, IL United States; ^4^ Children's Nutrition Research Center Agricultural Research Service United States Department of Agriculture Houston, TX United States; ^5^ Department of Pediatrics Baylor College of Medicine Houston, TX United States; ^6^ Section of Pediatric Diabetes and Endocrinology Department of Pediatrics Baylor College of Medicine and Texas Children's Hospital Houston, TX United States

**Keywords:** adolescence, type 1 diabetes, parenting

## Abstract

**Background:**

Supportive parent involvement for adolescents’ type 1 diabetes (T1D) self-management promotes optimal diabetes outcomes. However, family conflict is common and can interfere with collaborative family teamwork. Few interventions have used explicitly strengths-based approaches to help reinforce desired management behaviors and promote positive family interactions around diabetes care.

**Objective:**

The aim of this protocol was to describe the development of a new, strengths-based behavioral intervention for parents of adolescents with T1D delivered via a mobile-friendly Web app called Type 1 Doing Well.

**Methods:**

Ten adolescent-parent dyads and 5 diabetes care providers participated in a series of qualitative interviews to inform the design of the app. The 3- to 4-month pilot intervention will involve 82 parents receiving daily prompts to use the app, in which they will mark the diabetes-related strength behaviors (ie, positive attitudes or behaviors related to living with or managing T1D) their teen engaged in that day. Parents will also receive training on how to observe diabetes strengths and how to offer teen-friendly praise via the app. Each week, the app will generate a summary of the teen’s most frequent strengths from the previous week based on parent reports, and parents will be encouraged to praise their teen either in person or from a library of reinforcing text messages (short message service, SMS).

**Results:**

The major outcomes of this pilot study will include intervention feasibility and satisfaction data. Clinical and behavioral outcomes will include glycemic control, regimen adherence, family relationships and conflict, diabetes burden, and health-related quality of life.

**Conclusions:**

This strengths-based, mobile health (mHealth) intervention aims to help parents increase their awareness of and efforts to support their adolescents’ engagement in positive diabetes-related behaviors. If efficacious, this intervention has the potential to reduce the risk of family conflict, enhance collaborative family teamwork, and ultimately improve diabetes outcomes.

**Trial Registration:**

ClinicalTrials.gov NCT02877680; https://clinicaltrials.gov/ct2/show/NCT02877680 (Archived by WebCite at http://www.webcitation.org/6xTAMN5k2)

## Introduction

### Background and Rationale

Type 1 diabetes (T1D) is one of the most common pediatric chronic conditions [1]: between 2001 and 2009, the prevalence among people under 20 years of age in the United States approached 1 in 500 youths. Diabetes management demands include frequent daily blood glucose monitoring, precise insulin calculations and adjustments, multiple insulin administrations via injections or subcutaneous insulin pump, and consideration of nutritional intake and physical activity. Difficulty in adhering to these demanding self-management tasks contributes to suboptimal glycemic outcomes and increased risk for serious complications [2]. Difficulties with self-management and elevated glycemic outcomes are common during adolescence [3,4], when youths are often expected to take increasing responsibility for daily management [5,6]. Supportive parent involvement promotes optimal diabetes outcomes [7], yet maintaining positive, collaborative parent-adolescent interactions can be challenging in the context of normative adolescent development and diabetes-related stressors. Many families describe escalating conflict and difficulty in working together for T1D management [8,9], which increases the risk for poor clinical, behavioral, and glycemic outcomes [10,11]. Negative interactions stemming from parents’ frustration and fears about the consequences of poor glycemic control can interfere with positive family teamwork [9].

Several interventions for adolescents with T1D report improvements in family collaboration, youths’ quality of life (QOL), self-management behaviors, and prevention of deteriorations in glycemic outcomes [12,13]. Existing interventions often target behavioral risk factors for poor diabetes outcomes such as diabetes-related conflict, ineffective family management, and maladaptive coping [12]. Unfortunately, effect sizes compared with control groups are modest and in-range glycemic outcomes (ie, glycated hemoglobin A_1c_ [HbA_1c_] <7.5% [14]) are not consistently achieved or maintained [12].

*Diabetes Resilience* is defined as the achievement of optimal diabetes outcomes (ie, good QOL, high engagement in self-management behaviors, and close to target glycemic outcomes) despite the numerous challenges inherent to having T1D [15]. New interventions that use positive psychology strategies and aim to promote resilient outcomes in pediatric populations are gaining attention as an approach to extend the impact of existing behavioral interventions [16-19]. Strengths-based interventions for other populations have increased gratitude, happiness, and self-control; decreased psychological symptoms and behavior problems; and improved subjective well-being [20,21]. However, relatively few interventions promote optimal diabetes outcomes by explicitly reinforcing youth and family strengths [22], or positive diabetes-related attitudes and behaviors (eg, confidence, seeking support [15]) that have demonstrated associations with resilient outcomes [23,24].

Existing behavioral interventions are often multicomponent programs delivered by trained interventionists at multiple in-person sessions in the medical setting [12]. Time and space requirements for intervention delivery, families’ ability to attend multiple sessions, and the expenses of hiring and training behavioral interventionists limit the potential for widespread dissemination. Although behavioral support is consistently recommended by international diabetes care guidelines [14,25,26], few practices have the resources to employ behavioral care providers or routinely provide such services [27,28]. Limited resources must be prioritized for patients at the highest level of psychosocial need, leaving many without access to potentially helpful behavioral interventions [29].

Mobile health (mHealth) technologies address many of these limitations by reducing barriers to dissemination [30-32]. For example, intervention automation and delivery via mobile devices minimize the need for space, time, and specialized interventionists to deliver behavioral care [33-35]. Mobile health interventions for people with diabetes have included text message–based (short message service, SMS) reminders to perform diabetes self-management activities (eg, blood glucose monitoring, insulin administration, physical activity), automated reinforcement for engaging in self-management activities via text message, diabetes education via mobile phone or email, and guidance for making diabetes care decisions (eg, insulin dosing calculators, advice for managing low blood glucose); these interventions have generally been well received but have reported only modest results [35-38]. Previous mHealth interventions have largely targeted adolescents with T1D and adults with T1D or type 2 diabetes, but have not been delivered directly to parents of youths with T1D. Multicomponent, evidence-based behavioral interventions for adolescents with T1D and their parents have begun to be adapted for delivery via websites or mobile phone apps, and initial results suggest high acceptability and at least short-term improvements in adherence [36,39]. On the basis of previous research, recommendations for mHealth interventions in diabetes include minimizing demands and burden for participants (eg, patients, family members, providers), providing timely feedback about the targeted outcome, and relying on behavior change theory to inform the intervention/app design [35,38].

### Study Overview and Aims

This paper describes the study design and protocol for the Type 1 Doing Well pilot intervention study, which aims to address the need for brief, low-burden, and easily translatable behavioral interventions to facilitate positive family interactions and ultimately strengthen resilient diabetes outcomes during the challenging adolescent period. The purpose of the Type 1 Doing Well study is to develop and evaluate the feasibility of a strengths-based behavioral intervention for parents of adolescents with T1D, delivered via a mobile-friendly Web app that parents use for approximately 3 to 4 months, the standard period of time between quarterly diabetes clinic visits. The app uses a monitoring plus feedback design to help parents recognize and reinforce what their adolescents are doing well for diabetes management. Intervention components include the following: (1) *monitoring* by the app prompting parents to record observed strength behaviors (eg, asking an adult for help with insulin calculation, managing a difficult diabetes-related problem) their adolescents engage in each day; (2) *feedback* to parents by providing personalized weekly summaries of the adolescents’ most frequent strengths; and (3) *feedback* to adolescents by teaching and encouraging parents to reinforce their teens’ diabetes strengths. Theorized intervention mechanisms include enhancing protective behavioral and family processes by increasing parents’ awareness and reinforcement of diabetes actions that adolescents are doing well and facilitating positive family interactions in the context of everyday diabetes management, the ultimate goals of which are to improve adolescent QOL, self-management, and glycemic outcomes.

This protocol describes the process of developing and pilot testing the Type 1 Doing Well app. To develop the app, we employed user-centered design processes [40] to ensure the app would be engaging, easy to use, and meet families’ needs and preferences. The pilot intervention tests the resulting app with 82 adolescent-parent dyads. Each family will be randomized to either use the app (in addition to their usual diabetes care) or to a usual care control condition, in which they do not use the app, using a 2:1 randomization scheme. This exposes twice as many participants to the app; for a pilot study, this approach maximizes the amount of feedback received about the intervention to learn about its feasibility and acceptability, and generates more suggestions for refinement. The aims of the pilot intervention study are to evaluate the feasibility, acceptability, and preliminary impact of the intervention. Feasibility will be demonstrated by participant consent rates >70% and at least twice-weekly app use rates >75%. Acceptability will be demonstrated by high adolescent and parent satisfaction ratings >80%. To evaluate impact, it is hypothesized that adolescents will increase self-management behaviors and maintain or improve glycemic control pre- to postintervention. Because glycemic control often worsens during this period, either improvement or maintenance from baseline to follow-up is hypothesized to be better than the usual trend. It is also hypothesized that adolescents and parents will report improvements in diabetes strength behaviors, family conflict, diabetes burden, and health-related QOL from pre- to postintervention.

### Theoretical Framework

**Figure 1:**
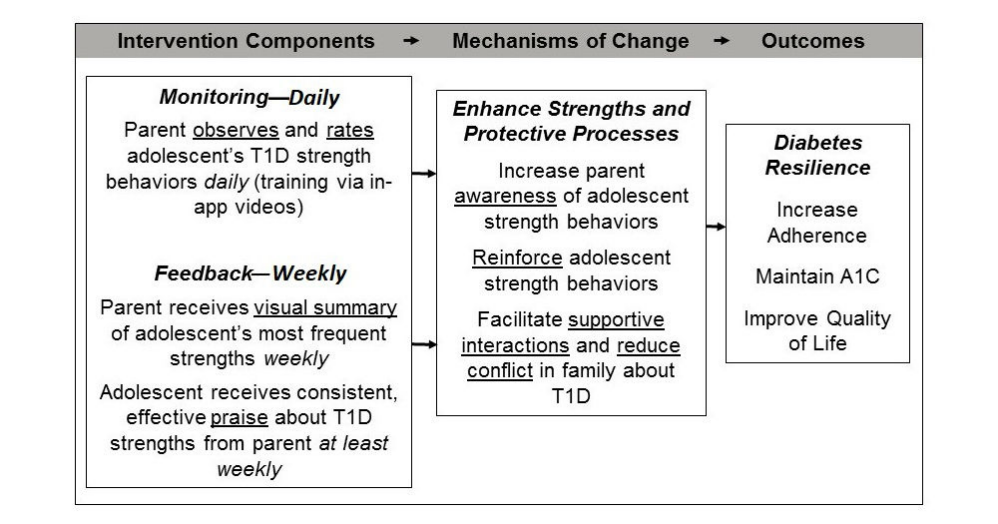
Conceptual model of strengths-based intervention components and theoretical mechanisms of change to reduce risk factors and promote resilient diabetes outcomes. T1D: type 1 diabetes; A_1c_: glycated hemoglobin A_1c_.

Type 1 Doing Well has its theoretical foundation in the Diabetes Resilience Model [15], which posits that diabetes outcomes are influenced by the individual’s and family’s risk factors and assets, and that enhancing positive protective processes that maximize diabetes-related strengths can buffer the negative impact of risks and amplify the positive impact of strengths, leading to resilient outcomes. By targeting positive family processes (eg, supportive parent involvement) and positive adolescent behaviors (eg, diabetes strengths: expressing confidence, seeking support), the Type 1 Doing Well intervention aims to enhance these protective factors, reduce the impact of risk factors (eg, diabetes burden, family conflict), and ultimately promote resilient diabetes outcomes (Figure 1). This approach represents a shift in tone from routine diabetes care (which by necessity focuses on identifying and solving problems with diabetes management) and from existing behavioral interventions (which seek to reduce behavioral barriers to optimal outcomes).

Compared with in-person interventions, a primary benefit of mHealth is ecological validity: data are collected and participants experience the intervention in the context of everyday activities [30,41,42]. Ecological validity can enhance intervention impact through increased proximity and relevance to the everyday behaviors being targeted and can reduce barriers to engagement. By making daily contact for strengths monitoring and weekly contact for feedback and to encourage parents to offer praise, this intervention is designed to increase their ability to apply strengths-based behavioral strategies in vivo. The focus on monitoring adolescents’ strengths rather than risks or problems throughout the day in this study may also increase the intervention’s appeal, and thus boost participant engagement, all noted challenges in existing interventions [21].

## Methods

### Design Overview

**Figure 2:**
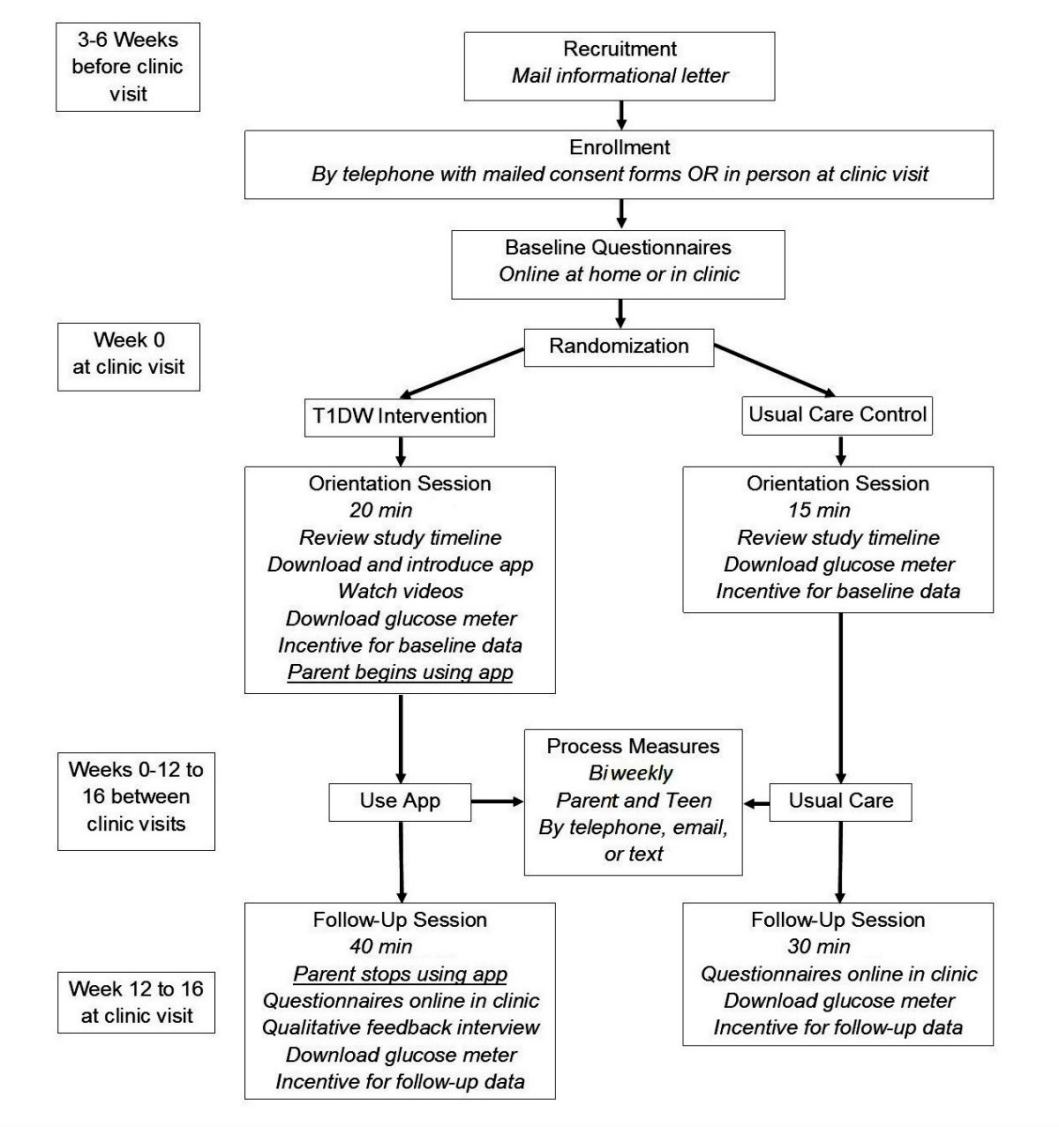
Schedule of participant activities. TIDW: Type 1 Doing Well.

Participants will be randomized 2:1 to the intervention and comparison groups, respectively, to maximize feasibility data and feedback from the intervention group. The intervention group will use the app for 3 to 4 months, between 2 outpatient diabetes clinic visits. The control group will receive usual care and will not use the app during the study period. Data collection will occur at baseline and follow-up for both the groups. The schedule of participant activities is outlined in Figure 2.

Participants in both the groups will receive US $25 per person for completing both baseline and follow-up questionnaires. They will also receive an additional US $5 per person for each biweekly survey they complete (described below). At each of 2 study visits (1: baseline data collection, randomization; 2: follow-up data collection), the family will receive US $13 to cover parking and/or transportation expenses. Adolescents will receive an additional US $5 per study visit for bringing all actively used blood glucose meters for download. Parents and adolescents in both the groups will also receive a small silicone wallet embossed with the study logo that adheres to the back of a mobile phone as an additional incentive, which also serves as a visual reminder to participants about the study. Because intervention feasibility is a primary study aim, incentives will be provided for completing data collection procedures but not for participation in the intervention. Intervention group participants will receive US $10 per month to offset data usage charges from using the app.

The protocol was approved by the institutional review board and is registered on ClinicalTrials.gov (NCT02877680). Data monitoring committees are not required for pilot studies; however, annual review by the institutional review board monitors for adverse participant events and conducts random audits. Protocol amendments are processed through the institutional review board, and significant changes are approved by the study sponsor.

### Recruitment

The target sample is 82 parent-adolescent dyads. Inclusion criteria are as follows: adolescent aged 12 to 17 years at enrollment, treated for T1D at the children’s hospital diabetes clinics where recruitment is taking place, and diabetes duration of at least 6 months. Parents (female or male) who self-identify as the primary caregiver will be eligible. Because the validated questionnaires and app are not available in other languages, fluency in English for parents and adolescents is required. Because the intervention is delivered via an app, parents must have a mobile device (ie, mobile phone or device that can receive text messages and access the Internet) with a data plan. Adolescents are not required to have a mobile device, as they will not be interacting directly with the app, and parents have the option to praise their adolescent by a text message or in person. Exclusions include serious medical, cognitive, or mental health comorbidity in parents or adolescents that would preclude the ability to participate. Recruitment takes place at 2 diabetes clinics serving patients of the Pediatric Diabetes and Endocrinology Service at a large tertiary children’s hospital affiliated with an academic medical center in the Southern United States. The 2 clinics were selected based on the size of the patient load at each clinic and to maximize study staff availability to be present for recruitment and study activities most days of the week. Following established diabetes clinic research processes, research staff review clinic schedules and mail informational letters to potentially eligible families and follow up by telephone call to introduce the study and conduct eligibility screening. Staff meet potentially eligible families in clinic for recruitment, informed consent, data collection, and enrollment. When possible, participants provide written consent and are sent a weblink to complete questionnaires before the clinic visit. When that is not possible, families are sent the weblink to complete questionnaires immediately following the in-clinic meeting, and participation in the intervention begins at the subsequent clinic visit. Families who decline participation will be asked to share the reason for choosing not to participate.

### Randomization

After consenting, enrolling in the study, and completing baseline questionnaires, participants will be randomized to the intervention or control group. A 2:1 randomization scheme with random block sizes will be used to maximize feasibility data and feedback from the intervention group (n=55 for intervention, n=27 for comparison). Following randomization, the intervention group will begin the intervention immediately. Research staff will help the parent install the app, register a username and password to access the app, and demonstrate app use. Staff will show the family 3 instructional videos within the app that teach participating parents about the importance of praise, how to identify strength behaviors, and how to provide developmentally appropriate praise to adolescents. Participants in this group will be instructed to begin using the app that day.

### Type 1 Doing Well Intervention: Development and Design

Semistructured qualitative interviews were conducted with 10 adolescents (mean age 15.4 [SD 1.9] years, 50% (5/10) female, 60% (6/10) non-Hispanic white, mean diabetes duration 7.0 [SD 3.8] years, 90% (9/10) privately insured) and their parents (70% (7/10) mothers) and 5 pediatric diabetes care providers (100% (5/5) physicians, 60% (3/5) faculty, 40% (2/5) postdoctoral fellows, 60% (3/5) female) to inform app design and intervention content. Parent and adolescent interviews asked about their perspectives on what positive diabetes-related behaviors adolescents tend to engage in and what adolescents wish their parents appreciated about their lives with diabetes, and how parents communicate with adolescents about diabetes management. Diabetes strengths described by parents and adolescents included adolescents performing diabetes self-management tasks without reminders, planning ahead for diabetes management during meals or activities, prioritizing diabetes management even when other events are occurring (eg, school, sports), using a positive tone during family communication about diabetes, and talking openly about diabetes with friends. Adolescents highlighted their desire for their parents to notice “small” everyday behaviors (eg, doing blood glucose checks), to provide specific rather than generic (eg, “good job”) praise, and to focus more on the adolescents’ efforts than on blood glucose values. The interviewer also sought parents’ and adolescents’ input on the app content and structure, including parents’ preferred frequency of contacts from/with the app, desired features of the app, for how long they might use the app, and recommendations to make the app appealing and usable. Feedback included asking parents to use the app no more than 1 to 2 times per day to rate their adolescents’ behavior over the previous 12 to 24 hours, and providing the strengths summary once a week (on the weekend) in a simple and easy-to-interpret list or graph, preferably using visually appealing colors. Parents and teens indicated that they communicate frequently via text message and thought that would be a useful way to provide praise. Some parents wanted the app to provide brief template praise texts that they could personalize and others wanted the option to create their own text messages. Adolescents were open to receiving texts from parents about what they did well and wanted the texts to be simple and straightforward, without adding comments about problems, concerns, or tasks that the adolescents needed to do. Several parents and adolescents noted that they would like to include emojis in the texts.

Diabetes care provider interviews focused on their observations of adolescents’ diabetes strengths and parent-adolescent interactions, and their perspectives on how the app could potentially be integrated into routine diabetes care practices. Strengths identified by providers included adolescents taking initiative to complete some diabetes tasks independently/with few parent reminders, using positive communications strategies with parents, seeking support from peers, and establishing diabetes self-management routines or habits. Their suggestions for the app included minimizing burden on app users by keeping the app format simple and the questions brief, introducing the app not as a punishment for poor parenting but rather as a tool for all parents, and having the option to add new strengths or text messages into the app. Providers highlighted the importance of helping parents focus on praising their adolescents more, and they suggested incorporating a summary of strengths-oriented data from the app into clinical conversations at later clinic visits.

After developing a prototype of the app based on this input, 4 families were shown screenshots of various features of the app, and they gave feedback (eg, ranked their preferences for strengths summary formats, commented on unclear or irrelevant strengths or text message templates). After finalizing the app design and components, research staff and software developers conducted extensive usability testing with the app to ensure optimal functionality.

**Figure 3:**
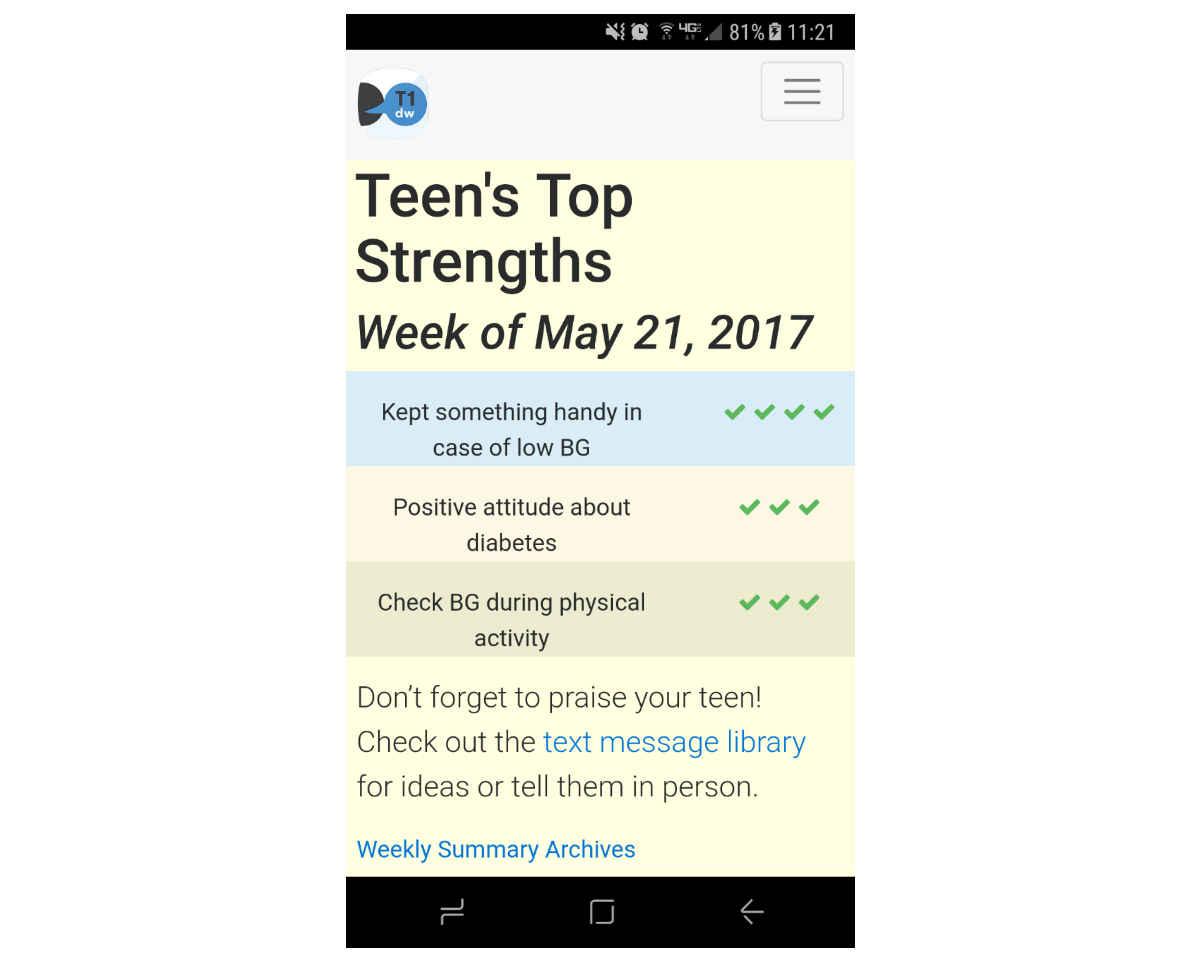
Screenshot of weekly strengths summary. BG: blood glucose.

On the basis of parent preferences and provider recommendations to limit parent burden in using the app, the app prompts parents by SMS and/or email (parent chooses method) to use the app once each day in the evening. A second prompt is sent 90 min later if the parent has not yet logged into the app. After logging in, parents are asked to select from a list of diabetes strength behaviors that their adolescent engaged in during the previous day. The strength behaviors were developed based on review of the literature and input from parents, adolescents, and providers about positive actions and attitudes that adolescents tend to engage in around diabetes self-management. Example strengths include the following: “Checked blood glucose without being asked,” “Discussed diabetes in a calm or positive way,” and “Took care of diabetes when he/she had a lot of other things going on.” In response to suggestions to incorporate new, personalized content to the app, if no response options apply, parents can select “other” and provide a written description of other positive behaviors observed, which are added to the list of strengths and can be selected by the parent again at a later time. Parents can also log into the app at other times and provide additional reports of their adolescent’s strength behaviors. Once a week, parents receive an additional prompt to view a summary of the top 3 strength behaviors they reported their adolescent engaged in over the previous week. On the basis of parent feedback, the summary is presented graphically as a list with checkmarks indicating how frequently each strength behavior was reported (Figure 3). The summary tab of the app links to a library of sample text messages that parents can copy into their mobile phone’s text messaging or email program to send to their child. The text wording was guided by adolescent suggestions. Parents have the option to edit the texts, write their own, or provide praise in person. In response to participant suggestions to include emojis, a collaboration was formed with Joyce Lee, MD MPH, who developed the “Diabetes Emoticons” emojis. Because the emoji pack can be used only on Apple devices, it could not be incorporated into the T1 Doing Well app (which was designed to be accessible on any device, described below). However, study staff inform participants that the emojis can be downloaded by Apple users and may be used to personalize their praise text messages, and the app includes a link to the free sticker pack in the iTunes store. The app also includes 3 videos described above, the content of which was informed by parent and adolescent input about which positive behaviors to track and how to offer praise in an adolescent-friendly way. The videos also address frequently asked questions (eg, how to respond when it is difficult to identify any positive behaviors). Parents watch these videos after being randomized to the intervention group and can access them at any time for a refresher.

The app was created and is maintained by faculty, staff, and software developers at the Center for Behavioral Intervention Technologies Development Core Facility at Northwestern University School of Medicine. To maximize the number of people who could use the app and not limit eligibility by platform (eg, Android vs Apple products), the app was created as a mobile-friendly website that is optimized for Android (with Chrome, Firefox, or Android v5.0+ Web browsers), iOS (with Chrome, Firefox, and Safari Web browsers), and Windows 10 (with Microsoft Edge) mobile devices and Mac (with Chrome, Firefox, Safari, and Opera Web browsers) and Windows (with Chrome, Firefox, IE10+, Microsoft Edge, and Opera Web browsers) desktop, laptops, and tablets. To increase app accessibility for participants, Android and iOS home screen app icons were developed so that users can save the icons to their home screen and access the Web app as if it were a mobile app. In addition to the participant-facing app, there is also a researcher-facing dashboard that allows research staff to add content (eg, new praise texts or strengths), manage participant access to the app and support requests, and export app usage data and participant summaries in real time.

### Usual Care Comparison Condition

Participants in both groups will receive usual care for T1D, which includes approximately quarterly visits with a multidisciplinary (ie, physician and advance practice nurse, nurse/medical assistant, registered dietician and certified-diabetes educator, and social worker) diabetes care team. The diabetes care team also includes a licensed clinical psychologist with expertise in diabetes who is available for treatment referrals based on provider concern or family request. Usual care without app use was selected as the comparison condition, given the early phase of development of the study.

### Measures

Data are collected at 2 times: parents and adolescents complete questionnaires at baseline following consent and before randomization, and at the diabetes clinic visit following randomization (approximately 3-4 months later). Questionnaires are collected via secure, HIPAA (Health Insurance Portability and Accountability Act)-compliant Web survey hosted through the institution’s clinical trials management system. Adolescents and parents have unique log-ins and passwords and are instructed to complete the surveys independently. Participants have the option to not answer any questions that they chose not to answer; if 3 or more questions are unanswered on a questionnaire, the system provides the following prompt: “You have not answered some questions in this survey. Click ok to continue or cancel to answer those questions.” Study data are identified with randomly generated participant numbers, and any paper materials are stored separately from documentation of identifiable health information, including informed consent.

To assess the process of the intervention’s impact, adolescents and parents each provide biweekly ratings of their relationship quality throughout the 3- to 4-month study period. These items can be answered via email, phone, or text message. Medical chart data are also collected during this period. Attempts are made to collect follow-up data from all participants, including those who stop using the app, to assess the impact of intervention dose.

#### Feasibility

Recruitment data will be used to calculate the percent of eligible families who enroll in the study. Usage data from the software platform (accessed via the research dashboard) will be used to calculate parent engagement with specific app features (eg, response rate to prompts, frequency of opening feedback summaries, frequency of using provided texts) and the percent of participants who interact with any app feature at least twice weekly.

#### Participant Characteristics

At baseline, parents will report their relationship to the adolescent, parent and adolescent’s racial and ethnic backgrounds, number of parents/caregivers living in the home, the adolescent’s insurance status (private, public, or none), and the highest level of parental education. Adolescents’ gender, date of birth (to calculate age), date of diabetes diagnosis (to calculate duration of diabetes), and current diabetes treatment regimen will be extracted from the medical record.

#### Clinical Diabetes Outcomes

Glycemic control (HbA_1c_) and blood glucose monitoring frequency are collected routinely at diabetes clinic visits and will be extracted from the electronic medical record. Trained phlebotomists draw blood samples by fingerstick and analyze assays immediately using the DCA 2000 Hemoglobin HbA_1c_ system (Siemens-Bayer). Blood glucose meters are routinely downloaded to calculate mean daily blood glucose monitoring frequency (previous 14 days).

#### Behavioral Outcomes

Adolescents self-report on the frequency of resilience-promoting attitudes and behaviors (ie, strengths) via the Diabetes Strengths and Resilience measure [23], a 12-item measure of perceived mastery over T1D management demands and accessing social/family support for T1D needs. They also complete the Monitoring Individual Needs In Diabetes (MIND)-Youth Questionnaire [43], a 33-item measure of diabetes health-related QOL. Parents rate youth engagement in diabetes self-management behaviors using the appropriate version (based on the youth’s insulin regimen) of the 24-item Diabetes Self-Management Profile Self-Report [44] and adolescents using the 15-item Self-Care Inventory-Revised [45].

#### Intervention Mechanisms

To assess the process of intervention impact, parents complete the PedsQL Family Impact Module (36 items) [46] and the Diabetes Family Impact Scale (14 items) [47], which measure the impact of diabetes on family activities and relationships and parent QOL. Diabetes-specific burden will be assessed via the Problem Areas in Diabetes measures for adolescents (26 items) [48] and parents (18 items) [49], both of which demonstrate good psychometric properties. Parents and youths complete the Diabetes Family Conflict Scale-Revised [50], a 19-item scale of family conflict surrounding diabetes issues, which has good reliability and validity. Parents and youths also complete the Helping for Health Inventory [51], a 15-item measure of unhelpful parental attention to the adolescent’s diabetes management. In addition to the questionnaire batteries at baseline and follow-up, every 2 weeks, parents and adolescents are asked to each rate their relationship quality using 3 items adapted from the Parent-Youth Relationship Index of the National Longitudinal Study of Youth-1997 [52].

#### Intervention Satisfaction and Feedback

Postintervention, parents in the intervention group complete the USE Questionnaire [53], a 32-item measure of the users’ perceived usefulness of, satisfaction with, and ease of use of a particular technology. Semistructured interviews with participants (separately with parents and adolescents) will be conducted to discuss experiences with the app (eg, intrusiveness of prompts, clarity/relevance of response options, usefulness and appeal of strength behavior feedback charts) and obtain suggestions for improvement.

### Planned Statistical Analyses

Intervention feasibility will be determined if the upper bound of the two-sided 95% exact, binomial CI is ≥95% for downloading the app and ≥70% for using the app at least twice per week. Acceptability will be measured as the proportion of participants who rate the intervention as acceptable (somewhat to very much) with 95% exact, binomial CIs (upper bound should be ≥80%).

Baseline participant demographics and clinical characteristics will be summarized with descriptive statistics, stratified by treatment group. Group differences will be assessed using two-sided, two-sample *t* tests, Wilcoxon rank sum tests, or Fisher exact test as appropriate. Statistical significance for univariate analysis will be assessed at the .05 level. However, this is a randomized clinical trial; therefore, significant group differences must be due to chance **,** and those variables will be adjusted for in a multiple regression model to confirm the magnitude of the effect.

The analysis of preliminary impact of this pilot intervention on the primary outcomes will compare mean blood glucose monitoring frequency, survey measures of self-management behaviors, and HbA_1c_ at follow-up between groups using an independent, two-sample *t* test. Equal variances will be assumed, unless the *F* test rejects the null hypothesis of equality at the .05 level. Quantile-quantile plots and Shapiro-Wilk tests, stratified by group, will be used to test for approximate normality, and data transformations (eg, natural logarithm) will be used if needed. Statistical significance will be assessed at the two-sided alpha=.05 level. A separate analysis will be used for each primary outcome **,** and no adjustments for multiple hypothesis tests will be made in this pilot study.

A general linear mixed model will also be used to compare follow-up measures between groups, adjusting for baseline response and baseline characteristics that are unequally distributed between groups. The generalized linear model will allow for the use of all available data, including participants with incomplete sets of observations. The model will include fixed effects for treatment group, time (discrete), baseline response, and a group-time interaction term. The model will also assume an unstructured matrix of correlated error terms to account for repeated observations. Statistical significance will be assessed at the .05 level for all hypothesis tests. All analyses will assume intention to treat. Missing data will not be imputed for the main analysis of primary or secondary outcomes. We anticipate very little missing data for these outcomes. Total scores will be calculated based on each measure’s scoring instructions (including instructions for handling missing item responses) and used in analyses. Analysis of secondary behavioral outcomes will be analyzed using a similar approach.

### Power

A total sample size of n=66 with complete data would be required to detect a standard effect size of 0.75 with 80% power using an independent, two-sample t test with equal variances assuming alpha=.05 and a 2:1 ratio between groups (n=44 for intervention, n=22 for control). This sample size will also have >95% probability of generating binomial CIs with half-widths ≤0.20. Therefore, we aim to enroll 82 (55 intervention, 27 control) participants to allow for a conservative 20% attrition rate.

## Results

Enrollment began in July 2017 and data collection will continue through June 2018. Data cleaning and analysis will be conducted following the completion of data collection. We anticipate reporting results in late 2018 through professional presentations and publications.

## Discussion

Parents’ understandable and valid concerns about the consequences of chronic poor glycemic control can heighten their attention to nonadherence [54,55], making it difficult to recognize adolescents’ positive self-management behaviors when they occur. The strengths-based Type 1 Doing Well intervention aims to focus parents’ attention on specific positive behaviors that their adolescents are engaging in, teach strategies to recognize adolescents’ strengths, and create frequent opportunities to offer reinforcement and praise. This explicit focus on identifying and reinforcing what adolescents are doing right has the potential to enhance supportiveness and collaboration in the parent-adolescent relationship around diabetes management, which are well-documented predictors of adherence and better glycemic control [7]. Training parents to consistently recognize and reinforce adolescents’ strength behaviors may also increase these desired behaviors, which have demonstrated associations with key diabetes outcomes [21,22]. This pilot study will evaluate the feasibility of implementing the mHealth intervention to parents of adolescents, parents’ and adolescents’ satisfaction with the intervention, and its preliminary impact on key behavioral and clinical outcomes.

Potential limitations of this study are primarily related to the pilot nature of the study design. Because the emphasis is on evaluating the feasibility and satisfaction of the intervention to inform future research, the sample size may not be large enough to detect small to moderate effects. The sample size was based on an estimated standard effect size of 0.75. This is higher than reported effect sizes in other behavioral mHealth and eHealth research with youths with chronic health conditions (*d* range=0.13-0.35 for change in health behaviors or disease control outcomes [56,57]) or positive psychology interventions (*d* range=0.20-0.34 [58]). However, mobile phone-based behavioral interventions targeting adults report a wide range of effect sizes, including some that are large (*d*=0.09-1.38 [59]). Moreover, relatively higher effect sizes have been reported in mHealth and eHealth interventions delivered to caregivers of youths with chronic medical conditions compared with those delivered to the youths themselves [56], and among those that incorporate theory-based behavior change strategies [60], as is the case for this study. In light of these effect size ranges, basing our power analysis on *d*=0.75 was deemed appropriate for a pilot study with a sufficient sample to conduct preliminary analyses of impact on key outcomes and estimate a plausible effect size for a larger study [61].

The use of a convenience sample at a single children’s hospital and the exclusion of non-English speaking participants may result in data that may not be representative of the population of families of adolescents with T1D. However, efforts are made to recruit a sample that includes parents and adolescents of both genders, adolescents spanning the eligible age range (12-17 years), and participants with diverse personal and clinical characteristics (eg, racial/ethnic background, insurance status, baseline HbA_1c_) to maximize potential generalizability of findings. Because the eligibility criteria for the study are not dependent on baseline glycemic control, self-management behaviors, or family functioning, it is possible that changes will not be able to be detected because of floor or ceiling effects at baseline. Because the app is accessed via a mobile website, it is not accessible when the device is out of the range of mobile data, which may limit participants’ use of the app in some situations. Conducting research in coordination with medical visits presents some challenges with scheduling and missed appointments. To minimize missing data at follow-up study visits, research staff remind all enrolled participants of upcoming scheduled clinic visits. However, some diabetes clinic visits do not occur at precise 3-month intervals. If a follow-up visit is not completed within 4 weeks of the 3-month follow-up window, a separate study visit is scheduled and glycemic data from the nearest clinic visit are used.

Promising results from this pilot study will inform the next phase of research to refine and further evaluate the effectiveness of this strengths-based mHealth intervention. The data gathered in this pilot study will comprise essential preliminary data to seek funding for evaluation in a larger, fully powered randomized controlled trial. In a larger trial, longer-term follow-up data will be collected to evaluate maintenance of improvements and/or additional improvements in outcomes over time. Ultimately, the goal is for this app to be used as a part of routine care of adolescents with T1D. Future research may include evaluation of this strengths-based app in combination with other mHealth technologies (eg, continuous glucose monitoring system platforms) or behavioral family interventions to enhance their impact on diabetes outcomes.

## Appendix

Multimedia Appendix 1 Peer-review report.

## Abbreviations

BG: blood glucose
HbA_1c_: glycated hemoglobin
mHealth: mobile health
QOL: quality of life
SMS: short message service
T1D: type 1 diabetes
TIDW: Type 1 Doing Well

## References

[1] Pettitt DJ, Talton J, Dabelea D, Divers J, Imperatore G, Lawrence JM, Liese AD, Linder B, Mayer-Davis EJ, Pihoker C, Saydah SH, Standiford DA, Hamman RF, SEARCH for Diabetes in Youth Study Group (2014). Prevalence of diabetes in U.S. youth in 2009: the SEARCH for diabetes in youth study. Diabetes Care.

[2] Hood KK, Peterson CM, Rohan JM, Drotar D (2009). Association between adherence and glycemic control in pediatric type 1 diabetes: a meta-analysis. Pediatrics.

[3] Hood KK, Beavers DP, Yi-Frazier J, Bell R, Dabelea D, Mckeown RE, Lawrence JM (2014). Psychosocial burden and glycemic control during the first 6 years of diabetes: results from the SEARCH for Diabetes in Youth study. J Adolesc Health.

[4] Rausch JR, Hood KK, Delamater A, Pendley JS, Rohan JM, Reeves G, Dolan L, Drotar D (2012). Changes in treatment adherence and glycemic control during the transition to adolescence in type 1 diabetes. Diabetes Care.

[5] Wiebe DJ, Chow CM, Palmer DL, Butner J, Butler JM, Osborn P, Berg CA (2014). Developmental processes associated with longitudinal declines in parental responsibility and adherence to type 1 diabetes management across adolescence. J Pediatr Psychol.

[6] Anderson BJ, Holmbeck G, Iannotti RJ, McKay SV, Lochrie A, Volkening LK, Laffel L (2009). Dyadic measures of the parent-child relationship during the transition to adolescence and glycemic control in children with type 1 diabetes. Fam Syst Health.

[7] Wysocki T, Nansel TR, Holmbeck GN, Chen R, Laffel L, Anderson BJ, Weissberg-Benchell J, Steering Committee of the Family Management of Childhood Diabetes Study (2009). Collaborative involvement of primary and secondary caregivers: associations with youths' diabetes outcomes. J Pediatr Psychol.

[8] Jaser SS (2011). Family interaction in pediatric diabetes. Curr Diab Rep.

[9] Spencer J, Cooper H, Milton B (2010). Qualitative studies of type 1 diabetes in adolescence: a systematic literature review. Pediatr Diabetes.

[10] Hilliard ME, Holmes CS, Chen R, Maher K, Robinson E, Streisand R (2013). Disentangling the roles of parental monitoring and family conflict in adolescents' management of type 1 diabetes. Health Psychol.

[11] Hilliard ME, Wu YP, Rausch J, Dolan LM, Hood KK (2013). Predictors of deteriorations in diabetes management and control in adolescents with type 1 diabetes. J Adolesc Health.

[12] Hood KK, Rohan JM, Peterson CM, Drotar D (2010). Interventions with adherence-promoting components in pediatric type 1 diabetes: meta-analysis of their impact on glycemic control. Diabetes Care.

[13] Hilliard ME, Powell PW, Anderson BJ (2016). Evidence-based behavioral interventions to promote diabetes management in children, adolescents, and families. Am Psychol.

[14] American Diabetes Association (2017). 12. Children and Adolescents. Diabetes Care.

[15] Hilliard ME, Harris MA, Weissberg-Benchell J (2012). Diabetes resilience: a model of risk and protection in type 1 diabetes. Curr Diab Rep.

[16] Rosenberg AR, Yi-Frazier JP, Eaton L, Wharton C, Cochrane K, Pihoker C, Baker KS, McCauley E (2015). Promoting resilience in stress management: a pilot study of a novel resilience-promoting intervention for adolescents and young adults with serious illness. J Pediatr Psychol.

[17] Kichler JC, Kaugars AS (2015). Applying positive development principles to group interventions for the promotion of family resilience in pediatric psychology. J Pediatr Psychol.

[18] Lord JH, Rumburg TM, Jaser SS (2015). Staying positive: positive affect as a predictor of resilience in adolescents with type 1 diabetes. J Pediatr Psychol.

[19] Hilliard ME, McQuaid EL, Nabors L, Hood KK (2015). Resilience in youth and families living with pediatric health and developmental conditions: introduction to the special issue on resilience. J Pediatr Psychol.

[20] Seligman ME, Steen TA, Park N, Peterson C (2005). Positive psychology progress: empirical validation of interventions. Am Psychol.

[21] Tolan P (2014). Future directions for positive development intervention research. J Clin Child Adolesc Psychol.

[22] Fogel NR, Weissberg-Benchell J (2010). Preventing poor psychological and health outcomes in pediatric type 1 diabetes. Curr Diab Rep.

[23] Hilliard ME, Iturralde E, Weissberg-Benchell J, Hood KK (2017). The diabetes strengths and resilience measure for adolescents with type 1 diabetes (DSTAR-Teen): validation of a new self-report measure. J Pediatr Psychol.

[24] Hilliard ME, Hagger V, Hendrieckx C, Anderson BJ, Trawley S, Jack MM, Pouwer F, Skinner T, Speight J (2017). Strengths, risk factors, and resilient outcomes in adolescents with type 1 diabetes: results from Diabetes MILES Youth–Australia. Diabetes Care.

[25] Pihoker C, Forsander G, Fantahun B, Virmani A, Luo X, Hallman M, Wolfsdorf J, Maahs DM, International Society for Pediatric and Adolescent Diabetes (2014). ISPAD Clinical Practice Consensus Guidelines 2014. The delivery of ambulatory diabetes care to children and adolescents with diabetes. Pediatr Diabetes.

[26] Young-Hyman D, de Groot M, Hill-Briggs F, Gonzalez JS, Hood K, Peyrot M (2016). Psychosocial care for people with diabetes: a position statement of the American Diabetes Association. Diabetes Care.

[27] Ducat L, Philipson LH, Anderson BJ (2014). The mental health comorbidities of diabetes. J Am Med Assoc.

[28] de Wit M, Pulgaron ER, Patino-Fernandez AM, Delamater AM (2014). Psychological support for children with diabetes: are the guidelines being met?. J Clin Psychol Med Settings.

[29] Fisher L, Glasgow RE (2007). A call for more effectively integrating behavioral and social science principles into comprehensive diabetes care. Diabetes Care.

[30] Schueller SM, Munoz RF, Mohr DC (2013). Realizing the potential of behavioral intervention technologies. Curr Dir Psychol Sci.

[31] Glasgow RE, Phillips SM, Sanchez MA (2014). Implementation science approaches for integrating eHealth research into practice and policy. Int J Med Inform.

[32] Bennett GG, Glasgow RE (2009). The delivery of public health interventions via the Internet: actualizing their potential. Annu Rev Public Health.

[33] Pulman A, Taylor J, Galvin K, Masding M (2013). Ideas and enhancements related to mobile applications to support type 1 diabetes. JMIR Mhealth Uhealth.

[34] Ben-Zeev D, Schueller SM, Begale M, Duffecy J, Kane JM, Mohr DC (2015). Strategies for mHealth research: lessons from 3 mobile intervention studies. Adm Policy Ment Health.

[35] Mulvaney SA, Ritterband LM, Bosslet L (2011). Mobile intervention design in diabetes: review and recommendations. Curr Diab Rep.

[36] Markowitz JT, Harrington KR, Laffel LMB (2013). Technology to optimize pediatric diabetes management and outcomes. Curr Diab Rep.

[37] Liang X, Wang Q, Yang X, Cao J, Chen J, Mo X, Huang J, Wang L, Gu D (2011). Effect of mobile phone intervention for diabetes on glycaemic control: a meta-analysis. Diabet Med.

[38] Herbert L, Owen V, Pascarella L, Streisand R (2013). Text message interventions for children and adolescents with type 1 diabetes: a systematic review. Diabetes Technol Ther.

[39] Thompson D, Cullen KW, Redondo MJ, Anderson B (2016). Use of relational agents to improve family communication in type 1 diabetes: methods. JMIR Res Protoc.

[40] De Vito Dabbs A, Myers BA, Mc Curry KR, Dunbar-Jacob J, Hawkins RP, Begey A, Dew MA (2009). User-centered design and interactive health technologies for patients. Comput Inform Nurs.

[41] Kaplan RM, Stone AA (2013). Bringing the laboratory and clinic to the community: mobile technologies for health promotion and disease prevention. Annu Rev Psychol.

[42] Mohr DC, Burns MN, Schueller SM, Clarke G, Klinkman M (2013). Behavioral intervention technologies: evidence review and recommendations for future research in mental health. Gen Hosp Psychiatry.

[43] de Wit M, Winterdijk P, Aanstoot H, Anderson B, Danne T, Deeb L, Lange K, Nielsen AO, Skovlund S, Peyrot M, Snoek F, DAWN Youth Advisory Board (2012). Assessing diabetes-related quality of life of youth with type 1 diabetes in routine clinical care: the MIND Youth Questionnaire (MY-Q). Pediatr Diabetes.

[44] Wysocki T, Buckloh LM, Antal H, Lochrie A, Taylor A (2012). Validation of a self-report version of the diabetes self-management profile. Pediatr Diabetes.

[45] Lewin AB, LaGreca AM, Geffken GR, Williams LB, Duke DC, Storch EA, Silverstein JH (2009). Validity and reliability of an adolescent and parent rating scale of type 1 diabetes adherence behaviors: the Self-Care Inventory (SCI). J Pediatr Psychol.

[46] Varni JW, Sherman SA, Burwinkle TM, Dickinson PE, Dixon P (2004). The PedsQL Family Impact Module: preliminary reliability and validity. Health Qual Life Outcomes.

[47] Katz ML, Volkening LK, Dougher CE, Laffel LM (2015). Validation of the Diabetes Family Impact Scale: a new measure of diabetes-specific family impact. Diabet Med.

[48] Weissberg-Benchell J, Antisdel-Lomaglio J (2011). Diabetes-specific emotional distress among adolescents: feasibility, reliability, and validity of the problem areas in diabetes-teen version. Pediatr Diabetes.

[49] Markowitz JT, Volkening LK, Butler DA, Antisdel-Lomaglio J, Anderson BJ, Laffel LM (2012). Re-examining a measure of diabetes-related burden in parents of young people with Type 1 diabetes: the Problem Areas in Diabetes Survey - Parent Revised version (PAID-PR). Diabet Med.

[50] Hood KK, Butler DA, Anderson BJ, Laffel LM (2007). Updated and revised Diabetes Family Conflict Scale. Diabetes Care.

[51] Harris MA, Antal H, Oelbaum R, Buckloh LM, White NH, Wysocki T (2008). Good intentions gone awry: assessing parental “miscarried helping” in diabetes. Fam Syst Health.

[52] Jones-Sanpei HA, Day RD, Holmes EK (2009). Core family process measures in the NLSY97: variation by gender, race, income, and family structure. Marriage Fam Rev.

[53] Lund AM (2001). ResearchGate.

[54] Leonard BJ, Garwick A, Adwan JZ (2005). Adolescents' perceptions of parental roles and involvement in diabetes management. J Pediatr Nurs.

[55] Ivey JB, Wright A, Dashiff CJ (2009). Finding the balance: adolescents with type 1 diabetes and their parents. J Pediatr Health Care.

[56] Fedele DA, Cushing CC, Fritz A, Amaro CM, Ortega A (2017). Mobile health interventions for improving health outcomes in youth: a meta-analysis. JAMA Pediatr.

[57] Cushing CC, Steele RG (2010). A meta-analytic review of eHealth interventions for pediatric health promoting and maintaining behaviors. J Pediatr Psychol.

[58] Bolier L, Haverman M, Westerhof GJ, Riper H, Smit F, Bohlmeijer E (2013). Positive psychology interventions: a meta-analysis of randomized controlled studies. BMC Public Health.

[59] Fjeldsoe BS, Marshall AL, Miller YD (2009). Behavior change interventions delivered by mobile telephone short-message service. Am J Prev Med.

[60] Webb TL, Joseph J, Yardley L, Michie S (2010). Using the internet to promote health behavior change: a systematic review and meta-analysis of the impact of theoretical basis, use of behavior change techniques, and mode of delivery on efficacy. J Med Internet Res.

[61] Czajkowski SM, Powell LH, Adler N, Naar-King S, Reynolds KD, Hunter CM, Laraia B, Olster DH, Perna FM, Peterson JC, Epel E, Boyington JE, Charlson ME (2015). From ideas to efficacy: the ORBIT model for developing behavioral treatments for chronic diseases. Health Psychol.

