# Supplementary material for: Strengths-Based Behavioral Intervention for Parents of Adolescents With Type 1 Diabetes Using an mHealth App (Type 1 Doing Well): Protocol for a Pilot Randomized Controlled Trial
Source: JMIR Res Protoc. 2018 Mar 13;7(3):e77. doi: 10.2196/resprot.9147 (PMC5871739; doi:10.2196/resprot.9147)
Supplement: Multimedia Appendix 1 [file resprot_v7i3e77_app1.pdf]

SUMMARY STATEMENT
(Privileged Communication)

PROGRAM CONTACT:
Christine Hunter
301-594-4728
hunterchristine@niddk.nih.gov

Release Date: 02/19/2016 12:59 PM
Revised Date:

Application Number: 1 R21 DK107951-01A1

Principal Investigator

HILLIARD, MARISA ELLEN

Applicant Organization: BAYLOR COLLEGE OF MEDICINE

Review Group: BMIO
Behavioral Medicine, Interventions and Outcomes Study Section

Meeting Date: 02/01/2016
Council: MAY 2016
Requested Start: 07/01/2016
RFA/PA: PA15-176
PCC: DKH BEHV

Project Title: Development and Pilot of a Strengths-Based Behavioral mHealth Intervention to Promote Resilience in Adolescents with Type 1 Diabetes
SRG Action: Impact Score:16
Next Steps: Visit http://grants.nih.gov/grants/next_steps.htm
Human Subjects: 30-Human subjects involved - Certified, no SRG concerns
Animal Subjects: 10-No live vertebrate animals involved for competing appl.
Gender: 1A-Both genders, scientifically acceptable
Minority: 1A-Minorities and non-minorities, scientifically acceptable
Children: 1A-Both Children and Adults, scientifically acceptable
Clinical Research - not NIH-defined Phase III Trial

Project Year	Direct Costs Requested	Estimated Total Cost
1	150,000	237,473
2	125,000	197,894
TOTAL	275,000	435,367

ADMINISTRATIVE BUDGET NOTE: The budget shown is the requested budget and has not been adjusted to reflect any recommendations made by reviewers. If an award is planned, the costs will be calculated by Institute grants management staff based on the recommendations outlined below in the COMMITTEE BUDGET RECOMMENDATIONS section.

1R21DK107951-01A1 Hilliard, Marisa

RESUME AND SUMMARY OF DISCUSSION: This application proposes to assess the feasibility and efficacy of a strengths-based behavioral mHealth intervention for parents of adolescents with type 1 diabetes. Both the environment and investigators are excellent. The investigators were highly responsive to prior critiques. During discussion a number of strengths were noted: inclusion of both qualitative and quantitative methods for intervention development, the involvement of parents, adolescents, and providers in the intervention development phase, the focus on a strengths-based intervention, and the use of near real time positive feedback to adolescents regarding self-care. In summary, the committee concluded that the potential overall impact was extremely high.

DESCRIPTION (provided by applicant): Type 1 diabetes (T1D) is among the most common chronic conditions of childhood and its management is complex and relentless. Adolescents have increased risk for worsening glycemic control, putting them at risk for short- and long-term complications. As responsibility for daily T1D management tasks begins to shift from parents to youth, supportive parent-adolescent teamwork promotes optimal diabetes outcomes. However, adolescents' cognitive development, desire for autonomy, and changing family and social relationships can make adherence to treatment recommendations difficult and strain attempts at parent-adolescent teamwork. We aim to develop and pilot test a mobile app-based behavioral intervention to facilitate positive, supportive parent-adolescent interactions around T1D management. The proposed study has two parts. First, we plan to involve adolescents with T1D (age 12-17), their parents, and diabetes care providers in designing a smartphone app that supports parents to recognize, keep track of, and reinforce their adolescents for specific positive T1D-related behaviors, or strengths. Example strengths include asking for help with complicated diabetes tasks, talking to friends about diabetes, and expressing confidence or optimism about T1D management. Intermittently throughout the day, the app will push parents a prompt to report which positive T1D behaviors their adolescent has engaged in. The app will generate weekly summary reports of each adolescent's most frequent strength behaviors, and parents will be reminded via the app to praise their adolescent for those patterns. Second, we plan to pilot test this intervention with 82 families; parents will be randomized to an intervention or a control condition. Participants in the intervention condition will use the app for 6 months and provide feedback, and control participants will receive usual care and will not use the app. Our main goal is to determine how often and in what ways families use the app, whether they like it, and to obtain suggestions for improvement. We will also evaluate trends for impact on important diabetes outcomes, such as quality of parent-adolescent relationships, T1D treatment adherence, and glycemic control. Data – including questionnaires, adherence data from blood glucose meters, and glycemic control biomarkers from a blood draw – will be collected at baseline and again 3 and 6 months later. The results of this pilot study will help refine the intervention so that it can be evaluated in a full- scale randomized controlled trial. Ultimately, the goal of this research is to validate brief, convenient, and helpful tools that families of all adolescents with T1D can use to strengthen positive family teamwork and ultimately promote optimal diabetes health outcomes.

PUBLIC HEALTH RELEVANCE: Type 1 diabetes (T1D) management is particularly challenging during adolescence as responsibility for management begins to shift from parents to youth, and positive family teamwork is critical to achieving optimal diabetes outcomes. Existing behavioral family interventions for T1D are beneficial but have limited potential for translation to clinical practice, and universal preventive approaches designed to explicitly promote existing T1D management strengths are needed. Ultimately, the goal of this line of research is to validate brief, convenient, and helpful tools that families of all adolescents with T1D can use to strengthen positive family teamwork and ultimately promote optimal diabetes health outcomes.

CRITIQUE 1

Significance: 2
Investigator(s): 1
Innovation: 2
Approach: 2
Environment: 1

Overall Impact: This R21 application addresses an interesting and important topic, improving adolescent self-care with a strengths-based mobile health intervention for parents and adolescents. The applicants were very responsive to prior review. They have a plan to include input from parents and adolescents into the development of the mobile app (Phase 1) and to then pilot test the app using a randomized controlled trial to assess feasibility and acceptability (Phase 2). Only the parent interview guide was included so the other interview guides could not be assessed. The pilot design is much improved and should lead to a better initial evaluation of the app. Minor issues exist with the data plan that can be easily corrected. The project has great potential for making an important contribution to the field.

1. Significance:

Strengths

- Adolescence in the presence of type 1 diabetes can be a turbulent period as the child struggles for autonomy. This period is typically associated with deterioration in glycemic control and often in parent-child relationships, putting the adolescent at risk. Thus, if successful, this project has the potential of advancing the field and impacting clinical care by taking advantage of the advances in technology.

Weaknesses

- None noted.

2. Investigator(s):

Strengths

- The investigative team is strong with well-known senior investigators participating. The team has strong skills in qualitative and quantitative measurements and analyses.
- The Northwestern Behavioral Intervention Technology Core site appears to have the necessary expertise to develop the mobile app. Dr. Joyce Ho Associate Director of the Center for Behavioral Intervention Technologies at Northwestern University has provided a letter of support.

Weaknesses

- None noted.

3. Innovation:

Strengths

- The strength based approach of the intervention is innovative in a parent and adolescent intervention and may be ideal to resolve several self-care and relationship issues centered on diabetes.

- Engaging adolescents and their parents in the app development process will help ensure that the app meets the needs of families with diabetes.
- Although the use of mobile technology for the app is not particularly innovative, it is very appropriate for this population and can extend the reach of the intervention.
- Using two rounds of interviews to help app development and app design is appropriate.

Weaknesses

The definition of resilience in terms of outcomes is somewhat unusual as it usually refers to a positive attitude accompanied by the ability to overcome significant barriers and is associated with self-efficacy and problem-solving skills. However, in the context of a strength-based intervention, the two definitions seem somewhat similar.

4. Approach:

Strengths

- The Investigators were responsive to prior reviews and have corrected the major weaknesses previously identified.
- Using interviews rather than focus groups is appropriate in both the app development phase and in the pilot and feasibility phase.
- The mixed method approach in the pilot and feasibility phase using both qualitative and quantitative techniques will lead to more comprehensive and complete data.

Weaknesses

- A comprehensive interview guide for parents was available but the adolescent version and the phase versions were not.
- It is not completely clear which members of the team will be analyzing the qualitative data thus it is not clear if this will be a multidisciplinary team.
- Randomization does not always work well when sample sizes are small thus attributing group differences to chance is inappropriate. Such differences should be thoroughly assessed. This is an easily corrected adjustment. Further, not correcting for multiple tests because it is a pilot study is not appropriate, although the whole issue is extremely controversial. Perhaps the goal is just to estimate effect sizes to inform the design of a larger study but reporting uncorrected levels of significance can mislead unknowing readers of the findings.
- Sensitivity to missing data in the models should be assessed using more modern techniques – using iterative analysis with multiple imputations with multiple generated data sets, not necessarily using the imputed results to report findings.

5. Environment:

Strengths

- The environment at Texas Children's is excellent. The Northwestern BITcore site also appears to have a strong research orientated environment. Both institutions have the necessary resources and patient population to support this work.

Weaknesses

- None noted.

Protections for Human Subjects:

Acceptable Risks and/or Adequate Protections

- This investigators identified possible risks in this minimal risk study and have included a plan to address these.

Data and Safety Monitoring Plan (Applicable for Clinical Trials Only):

Acceptable

- o A plan is included that details assessing study activities at weekly study meetings. The PI will take responsibility for identifying problem areas and including adverse events and will report all to the IRB.

Inclusion of Women, Minorities and Children:

- Sex/Gender: Distribution justified scientifically
- Race/Ethnicity: Distribution justified scientifically
- Inclusion/Exclusion of Children under 21: Including ages < 21 justified scientifically
- 85% of parents will be female; the sample will be 74% non-Hispanic White, 24% Hispanic, 15% African American, 9% more than one race; 4% Asian or American Indian One half of the children will be female; the children's races reflect those on the parents' table. Providers: 60% female; 40% NonHispanic White; 20% Hispanic; 20% Asian.

Vertebrate Animals:

Not Applicable (No Vertebrate Animals)

Biohazards:

Not Applicable (No Biohazards)

Revision:

- The application demonstrates responsiveness to the prior review. The investigators appear to have addressed every point raised.

Budget and Period of Support:

Recommend as Requested

CRITIQUE 2

Significance: 1
Investigator(s): 1
Innovation: 2
Approach: 2
Environment: 1

Overall Impact: This is a resubmission of an R21 application that proposes refinement and preliminary evaluation of the efficacy of a smartphone app designed to encourage parents of adolescents with type 1 diabetes to recognize, acknowledge and praise therapeutic self management behaviors exhibited by their adolescents. The revision is exceptionally responsive to the prior critiques and a number of key methodological, conceptual and analytic refinements have been made. These changes have further strengthened an already solid application. The potential impact of the proposed work is definitely high.

1. Significance:

Strengths

- Adequate self management of type 1 diabetes is a struggle for many teens and their parents.
- Changing this common developmental trajectory could prevent both acute and chronic complications of diabetes as well as maintaining more favorable quality of life.
- A focus on increasing parents' positive reinforcement of good self management behavior in their teens is a very plausible mechanism for achieving behavior change in a way that could be naturally self-sustaining.
- A clear definition is given of resilience and its place in the conceptual basis for the proposed intervention.

Weaknesses

- None.

2. Investigator(s):

Strengths

- A highly capable PI supported by a team of skilled, productive and experienced colleagues.

Weaknesses

- None.

3. Innovation:

Strengths

- Focus on promotion of adolescents' self management strengths via a smartphone app is a prudent objective.

Weaknesses

- Until it is produced and tested, there will always be doubt regarding whether the planned app can be developed to function as anticipated.

4. Approach:

Strengths

- Decreased time and effort dedicated to the qualitative piece is a prudent use of the short available time and funds.
- Change in study design to a straight up 6 month 2 group RCT with 2:1 randomization to the experimental intervention is cleaner, simpler than the design previously proposed.

- Addition of an enrollment criterion that ensures suboptimal adherence among enrolling adolescents is prudent. Less risk of diluted treatment effects due to enrollment of teens with near-optimal baseline management of diabetes.
- Clearer and more complete qualitative and quantitative analysis plans have been put forth.
- Greater assurance has been provided that BIT Core staff at Northwestern can in fact develop the app per the team's specifications.

Weaknesses

- Unclear why enrollment criteria do not focus on ensuring clinically meaningful problems with HbA1c or QOL since these are the primary outcomes.

5. Environment:

Strengths

- Very strong environment provides solid assurance that the research strategy can be carried out as proposed.

Weaknesses

- None.

Protections for Human Subjects:

Acceptable Risks and/or Adequate Protections

- A stronger human subjects protection plan has been outlined, including attention to risks of deterioration in the control group.

Data and Safety Monitoring Plan (Applicable for Clinical Trials Only):

Acceptable

- o An internal data and safety monitoring team will perform this function. Although this is a clinical trial, it poses minimal risk to participants.

Inclusion of Women, Minorities and Children:

- Sex/Gender: Distribution justified scientifically
- Race/Ethnicity: Distribution justified scientifically
- Inclusion/Exclusion of Children under 21: Including ages < 21 justified scientifically
- Of 92 youth (10 qualitative; 82 RCT) gender distribution is equal M:F; All are children; Racial minorities ~29% of sample; Hispanic ~23% of sample. All of these estimate are acceptable. Similar estimates for parents.

Vertebrate Animals:

Not Applicable (No Vertebrate Animals)

Biohazards:

Not Applicable (No Biohazards)

Resubmission:

- The resubmission has been highly responsive to the earlier critiques. Each comment is enumerated and a satisfactory change has been incorporated in response to each.

Budget and Period of Support:

Recommend as Requested

CRITIQUE 3

Significance: 2
Investigator(s): 1
Innovation: 1
Approach: 2
Environment: 1

Overall Impact: This R21 application proposes to design and then test the feasibility and initial efficacy of a strengths-based behavioral mHealth intervention for parents of adolescents with type 1 diabetes. The rationale is that a behavioral intervention focused on strengths-monitoring-plus-feedback to adolescents will result in better diabetes resilience (e.g., HbA1c, decreased diabetes burden, improved quality of life). The research team has been very responsive to reviewer comments and a stronger proposal has emerged as a result. The proposed intervention is significant because it is well-known that many adolescents with type 1 diabetes are at risk for worsening diabetes management and glycemic control. There is also evidence demonstrating increased conflict and decreasing parental support for diabetes during adolescence. Potential innovations include the inclusion of both qualitative and quantitative methods for intervention development, the involvement of parents, adolescents, and providers in the intervention development phase, the focus on a strengths-based intervention, and the opportunity for near real time positive feedback to adolescents regarding their self-care. Potential considerations for the Approach include extending or eliminating the proposed one-week trial of the app outlined in Phase 1. Given the frequency of text to parents (1-3 times per day), summary of adolescents strengths (1 time per week), and prompts to parents to praise their adolescent (1 time per week), it is likely a one week trial may not yield sufficient data to properly evaluate usability concerns or changes to the app. Another consideration is to include feedback from adolescents to determine if they recognized the addition of positive parental feedback and found this motivating. Otherwise this is a strong application and the impacts of this well-constructed study are expected to be high.

Protections for Human Subjects:

Acceptable Risks and/or Adequate Protections

- Adequate consideration of risks and protections.

Data and Safety Monitoring Plan (Applicable for Clinical Trials Only):

Acceptable

- One consideration, the proposed schedule of weekly meetings to review adverse events may not be frequent enough to allow for reporting to the IRB within three business days, as specified in the plan.

Inclusion of Women, Minorities and Children:

- Sex/Gender: Distribution justified scientifically
- Race/Ethnicity: Distribution justified scientifically
- Inclusion/Exclusion of Children under 21: Including ages < 21 justified scientifically
- Anticipate balanced sex/gender distribution for adolescents while mostly mothers are expected as parent participants (85%); Anticipate the full sample will be 50% Non-Hispanic White, 15% African American, 21% Hispanic/Latino, 5% other race/ethnicity and 9% multiple race.

Vertebrate Animals:

Not Applicable (No Vertebrate Animals)

Biohazards:

Acceptable

Budget and Period of Support:

Recommend as Requested

THE FOLLOWING SECTIONS WERE PREPARED BY THE SCIENTIFIC REVIEW OFFICER TO SUMMARIZE THE OUTCOME OF DISCUSSIONS OF THE REVIEW COMMITTEE, OR REVIEWERS' WRITTEN CRITIQUES, ON THE FOLLOWING ISSUES:

PROTECTION OF HUMAN SUBJECTS (Resume): ACCEPTABLE

INCLUSION OF WOMEN PLAN (Resume): ACCEPTABLE

INCLUSION OF MINORITIES PLAN (Resume): ACCEPTABLE

INCLUSION OF CHILDREN PLAN (Resume): ACCEPTABLE

COMMITTEE BUDGET RECOMMENDATIONS: The budget was recommended as requested.

Footnotes for 1 R21 DK107951-01A1; PI Name: Hilliard, Marisa Ellen

NIH has modified its policy regarding the receipt of resubmissions (amended applications). See Guide Notice NOT-OD-14-074 at http://grants.nih.gov/grants/guide/notice-files/NOT-OD-14-074.html. The impact/priority score is calculated after discussion of an application by averaging the overall scores (1-9) given by all voting reviewers on the committee and multiplying by 10. The criterion scores are submitted prior to the meeting by the individual reviewers assigned to an application, and are not discussed specifically at the review meeting or calculated into the overall impact score. Some applications also receive a percentile ranking. For details on the review process, see http://grants.nih.gov/grants/peer_review_process.htm#scoring.

MEETING ROSTER
Behavioral Medicine, Interventions and Outcomes Study Section
Risk, Prevention and Health Behavior Integrated Review Group
CENTER FOR SCIENTIFIC REVIEW

BMIO
02/01/2016 - 02/02/2016

CHAIRPERSON(S)

PALERMO, TONYA M, PHD
PROFESSOR
DEPARTMENT OF ANESTHESIOLOGY
AND PAIN MEDICINE SEATTLE
CHILDREN'S HOSPITAL RESEARCH INSTITUTE
UNIVERSITY OF WASHINGTON
SEATTLE, WA 98145

MEMBERS

AIKENS, JAMES E, PHD
ASSOCIATE PROFESSOR
DEPARTMENT OF FAMILY MEDICINE
UNIVERSITY OF MICHIGAN
ANN ARBOR, MI 48109

BOND, DALE S, PHD
ASSOCIATE PROFESSOR
WEIGHT CONTROL
AND DIABETES RESEARCH CENTER
THE MIRIAM HOSPITAL
BROWN ALPERT MEDICAL SCHOOL
PROVIDENCE, RI 02903

FILLINGIM, ROGER B, PHD *
DISTINGUISHED PROFESSOR
DEPARTMENT OF COMMUNITY DENTISTRY
AND BEHAVIORAL SCIENCES
COLLEGE OF DENTISTRY
UNIVERSITY OF FLORIDA
GAINESVILLE, FL 32610

HAMILTON, ROY H, MD
ASSISTANT PROFESSOR
DEPARTMENT OF NEUROLOGY AND
PHYSICAL MEDICINE AND
REHABILITATION HOSPITAL
UNIVERSITY OF PENNSYLVANIA
PHILADELPHIA, PA 19104

HOMMEL, KEVIN, PHD
ASSOCIATE PROFESSOR OF PEDIATRICS
CENTER FOR ADHERENCE
AND SELF-MANAGEMENT CINCINNATI
CHILDREN'S HOSPITAL MEDICAL CENTER
UNIVERSITY OF CINCINNATI
CINCINNATI, OH 45229

HOVELL, MELBOURNE F, PHD
DISTINGUISHED PROFESSOR AND DIRECTOR
CENTER FOR BEHAVIORAL EPIDEMIOLOGY
AND COMMUNITY HEALTH
SAN DIEGO STATE UNIVERSITY
SAN DIEGO, CA 92123

HURWITZ, ERIC L, PHD *
PROFESSOR
DEPARTMENT OF PUBLIC HEALTH SCIENCES
JOHN A BURNS SCHOOL OF MEDICINE
UNIVERSITY OF HAWAII, MANOA
HONOLULU, HI 96822

JENSEN, MARK P, PHD
PROFESSOR AND VICE CHAIR FOR RESEARCH
DEPARTMENT OF REHABILITATION MEDICINE
UNIVERSITY OF WASHINGTON MEDICAL CENTER
SEATTLE, WA 98195

KRULL, KEVIN R, PHD
PROFESSOR AND MEMBER
DEPARTMENT OF EPIDEMIOLOGY
AND CANCER CONTROL
ST JUDE CHILDREN'S
RESEARCH HOSPITAL
MEMPHIS, TN 38105

MA, JUN, MD, PHD
PROFESSOR
HEALTH POLICY AND ADMINISTRATION
DEPARTMENT OF MEDICINE
SCHOOL OF PUBLIC HEALTH
UNIVERSITY OF ILLINOIS AT CHICAGO
CHICAGO, IL 60608

MCLEAN, SAMUEL A, MD *
ASSOCIATE PROFESSOR
AND VICE CHAIR OF RESEARCH
DEPARTMENT OF ANESTHESIOLOGY
UNIVERSITY OF NORTH CAROLINA
CHAPEL HILL, NC 27599

OLOMU, ADE B, MD, MS
PROFESSOR AND VICE CHAIR FOR RESEARCH
DEPARTMENT OF MEDICINE
MICHIGAN STATE UNIVERSITY
EAST LANSING, MI 48824

PA, JUDY, PHD *
ASSISTANT PROFESSOR
LABORATORY OF NEURO IMAGING
STEVENS NEUROIMAGING AND INFORMATICS INST
KECK SCHOOL OF MEDICINE
UNIVERSITY OF SOUTHERN CALIFORNIA
LOS ANGELES, CA 90032

PARK, CRYSTAL L, PHD
PROFESSOR
DEPARTMENT OF PSYCHOLOGY
UNIVERSITY OF CONNECTICUT
STORRS, CT 06269

PATTON, SUSANA R, PHD *
ASSOCIATE PROFESSOR
DEPARTMENT OF PEDIATRICS
UNIVERSITY OF KANSAS MEDICAL CENTER
KANSAS CITY, KS 66160

PENEDO, FRANK J, PHD
PROFESSOR
DEPARTMENT OF MEDICAL
SOCIAL SCIENCES
FEINBERG SCHOOL OF MEDICINE
NORTHWESTERN UNIVERSITY
CHICAGO, IL 60611

PERRY, TAMARA TAYLOR, MD *
ASSOCIATE PROFESSOR
DEPARTMENT OF PEDIATRICS
COLLEGE OF MEDICINE
UNIVERSTIY OF ARKANSAS FOR MEDICAL SCIENCE
LITTLE ROCK, AR 72202

RAVENELL, JOSEPH E, MD
ASSISTANT PROFESSOR
DEPARTMENT OF POPULATION HEALTH
SCHOOL OF MEDICINE
NEW YORK UNIVERSITY
NEW YORK , NY 10016

RIEKERT, KRISTIN A, PHD
ASSOCIATE PROFESSOR
DIVISION OF PULMONARY
AND CRITICAL CARE MEDICINE
JOHNS HOPKINS SCHOOL OF MEDICINE
BALTIMORE, MD 21224

RODRIGUE, JAMES R, PHD
PROFESSOR AND VICE CHAIRMAN
DEPARTMENT OF SURGERY
BETH ISRAEL DEACONESS
MEDICAL CENTER
BOSTON, MA 02215

SEPHTON, SANDRA E, PHD *
ASSOCIATE PROFESSOR
DEPARTMENT OF PSYCHOLOGICAL
AND BRAIN SCIENCES
JAMES GRAHAM BROWN CANCER CENTER
UNIVERSITY OF LOUISVILLE
LOUISVILLE, KY 40292

WEINGER, KATIE, EDD
ASSOCIATE PROFESSOR OF PSYCHIATRY
JOSLIN DIABETES CENTER
HARVARD UNIVERSITY
BOSTON, MA 02215

WYSOCKI, TIM, PHD
CO-DIRECTOR
CENTER FOR HEALTH CARE DELIVERY SCIENCE
NEMOURS CHILDREN'S HEALTH SYSTEM
JACKSONVILLE, FL 32207

MAIL REVIEWER(S)

HOULE, TIMOTHY T, PHD
ASSISTANT PROFESSOR
ANAESTHESIA-MASSACHUSETTS GENERAL HOSPITAL
MASSACHUSETTS GENERAL HOSPITAL
BOSTON, MA 02114

SCIENTIFIC REVIEW OFFICER

MANN, LEE S, PHD
SCIENTIFIC REVIEW OFFICER
CENTER FOR SCIENTIFIC REVIEW
NATIONAL INSTITUTES OF HEALTH
BETHESDA, MD 20892

EXTRAMURAL SUPPORT ASSISTANT

JENNINGS, CHRISOULA, BS
LEAD EXTRAMURAL SUPPORT ASSISTANT
RISK, PREVENTION AND HEALTH BEHAVIOR
INTEGRATED REVIEW GROUP
CENTER FOR SCIENTIFIC REVIEW
NATIONAL INSTITUTES OF HEALTH
BETHESDA, MD 20892

* Temporary Member. For grant applications, temporary members may participate in the entire meeting or may review only selected applications as needed.

Consultants are required to absent themselves from the room during the review of any application if their presence would constitute or appear to constitute a conflict of interest.
